# The Outer Retinal Membrane Protein 1 Could Inhibit Lung Cancer Progression as a Tumor Suppressor

**DOI:** 10.1155/2021/6651764

**Published:** 2021-02-17

**Authors:** Min Zhang, Jun Jiang, Cui Ma

**Affiliations:** ^1^Department of Respiratory Medicine, The Second Affiliated Hospital of Zhejiang University School of Medicine, 88 Jiefang Road, 310009 Hangzhou, Zhejiang, China; ^2^State Key Laboratory of Genetic Engineering, School of Life Sciences, Fudan University, Shanghai 200438, China; ^3^Department of Endocrinology, Zhejiang Greentown Cardiovascular Hospital, 409 Gudun Road, 310012 Hangzhou, Zhejiang, China

## Abstract

Some related reports indicate that the outer retinal membrane protein 1 (ROM1) functions importantly in the regulation of the biological process of tumor. Nevertheless, studies towards the role of ROM1 in lung cancer are few. Here, our data demonstrated that ROM1 displayed a relation with lung cancer tumorigenesis and development. In the Tumor Genome Atlas (TCGA) cohort, reduced ROM1 level was observed in lung cancer tissues, instead of normal tissues. After bioinformatics analysis, the data revealed that ROM1 level was associated with the tumor stage. Additional results indicated that highly expressed ROM1 exhibited a positive correlation with the overall survival rate, and ROM1 was probably a promising prognostic biomarker of lung cancer. Additionally, our results indicated that knocking out ROM1 could promote cell proliferation, migration, and invasion. Our data conclusively demonstrated that ROM1 modulated lung cancer tumorigenesis and development, as a prognosis and treatment biomarker.

## 1. Background

In China, lung cancer is a widely occurring cancer and the primary cause of cancer-associated mortality. In 2015, there were approximately 733,000 newly generated lung cancer cases and 610,000 deaths [1]. Statistics illustrated that the ratio of non-small-cell lung cancer (NSCLC) patients took up 80% to 85% over total types of lung carcinoma [2, 3], and 30% of NSCLC patients entered stage III at the time of diagnosis and missed the best opportunity for surgical treatment. Stage III NSCLC is highly heterogeneous. The five-year survival rates in stages IIIA, IIIB, and IIIC NSCLC reach up to 36%, 26%, and 13%, respectively [4]. Surgical intervention is the most suitable for early lung cancer diagnosis and thought to be the best treatment option [5]. Numerous surgical interventions are being performed in patients, comprising wedge resection, segmental resection, lobectomy, and lung resection [6, 7]. In the past decade, much progress in treatment and diagnosis, such as immunotherapy and tumor genomics, has made a huge improvement in the survival of lung cancer [8, 9]. Lung cancer has become a successful example of molecular targeted therapy in solid tumors [10].

As a regulatory molecule, ROM1 plays an important role in human tumors in biological activities. ROM1 mRNA has been reported to participate in many cancers' progressions [11]. In both human and murine species, the coding region of ROM1 is included in about 1.8 kb of genomic DNA and is only separated by two introns. The splice sites of introns are well conserved [12]. Transcriptome data analysis shows that ROM1 can be used as a predictor for retinoblastoma progression [13]. The ablation of ROM1 leads to a change from the macular/pattern dystrophy (MD/PD) phenotype characterized by a defect in cone function to a retinitis pigmentosa (RP) phenotype characterized by a dominant defect in rod function, and the formation of abnormal Prph2/ROM1 complex and total Prph2 protein decreased. Therefore, ROM1 acts as a disease modifier by promoting the huge variability of PRPH2 related to disease phenotypes [14]. Bioinformatics analysis of the transcriptome data of clear cell renal cell carcinoma (ccRCC) reveals that the ROM1 may be an important candidate gene for understanding the molecular mechanism of ccRCC and can be used as a therapeutic target and diagnostic organism for ccRCC landmark [15]. The clinical feature and biological function of ROM1 in lung cancer are yet elusive.

Herein, we studied ROM1 expression and function in lung cancer through multiple bioinformatics analyses and related experimental data. Our data indicated that lowly expressed ROM1 was shown in lung cancer cells and its reduction could induce cancer progression. All the above-mentioned findings are helpful for lung cancer treatment.

## 2. Materials and Methods

### 2.1. Public Data Set

Three data sets were analyzed here. The first was The Cancer Genome Atlas (TCGA, http://gdac.broadinstitute.org); we obtained lung adenocarcinoma (LUAD) and lung squamous cell carcinoma (LUSC) RNA sequencing data and clinical assessment of cancer patients from TCGA. The second was the Gene Expression Omnibus (GEO, http://www.ncbi.nlm.nih.gov/geo/), containing GSE4573, GSE31210, GSE50081, GSE37745, and GSE30219. The third was cancer microarray (CaArray) informatics. CaArray developed by the National Cancer Institute was a widely used source, web, and programmable access array data management system.

### 2.2. GO and KEGG Pathway Analysis

The database was used to identify target genes for annotation, visualization, and comprehensive discovery (DAVID v6.8; https://david.ncifcrf.gov), based on gene ontology (GO) gene expression function annotation, pathway enrichment analysis, and Kyoto Encyclopedia of Genes and Genomes (KEGG) database. A cut-off value of *P* < 0.05 was used.

### 2.3. Clinical Specimens

The tissue samples were retrieved from the Second Affiliated Hospital of Zhejiang University School of Medicine. The samples had complete clinicopathological information, including 18 lung cancer tissues and 15 normal lung tissues. Our study got the approval of the Ethics Committee of this hospital.

### 2.4. Cell Culture

Human lung cancer cells comprising H1299 (CRL-5803), A549 (CCL-185), NCI-H460 (HTB-177), HCC827 (CRL-2868), NCI-H292 (CRL-1848), and normal lung cell BEAS-2B (CRL-9609) were acquired from American Type Culture Collection (ATCC; Manassas, VA, USA). All cancer cells were kept in RPMI-1640 medium (BI, Israel) with 10% FBS under a 37°C incubator containing 5% CO_2_. BEAS-2B was cultured in BEBM medium (BI, Israel) with 10% FBS.

### 2.5. SiRNA Transfection

SiRNAs were ordered from Shanghai Gene Pharmaceutical Co., Ltd. (Shanghai Gene Pharmaceutical Co., Ltd., China). Lipofectamine®RNAiMAX reagent (Thermo Fisher Scientific, Inc.) was applied to deliver 5 *μ*l siRNA for each well into indicated cells in the prepared six-well plate as the manual described. The sequence of siRNA was as follows: si-ROM1, 5′-GCAATGTAGAAGGCCTATA-3′; si-NC, 5′-UUCUCCGAACGUGUCACGUTT-3′.

### 2.6. RNA Extraction

TRIzol reagent was from Invitrogen, USA, and it was employed to harvest the whole RNA from above-mentioned cancer cells referring to the protocol. CDNA was synthesized by transcribing RNA.

### 2.7. Quantitative Reverse Transcription PCR (qRT-PCR)

RT-PCR was performed and described as follows: 15 minutes at 37°C and 5 seconds at 85°C on the LightCycler 96 RT-PCR instrument (Roche Diagnostics, Basel, Switzerland). qPCR reaction was conducted and described as follows: 95°C for 300 s, 40 cycles for 95°C for 15 s, 56°C for 15 s, 72°C for 15 s, then 95°C for 10s, 65°C for 60s, 97°C for 1 s, and then 37°C for 30 s in the cooling step. The relative gene expression was detected by SYBR-Green-PCR-Master Mix (Vazyme, Nanjing, China). *β*-Actin was used as internal control. The RNA primers were as follows: ROM1, 5′-GCCACGGGTACAAGGATTGG-3′ (forward), 5′-GGCCTTCTACATTGCTCTGGA-3′ (reverse); GAPDH, 5′-GGAGCGAGATCCCTCCAAAAT-3′ (forward), 5′-GGCTGTTGTCATACTTCTCATGG-3′ (reverse).

### 2.8. Cell Proliferation Assay

At posttransfection, cells were reseeded into 96-well plates and kept in RPMI-1640 medium with 10% FBS. The Cell Counting Kit (CCK-8) kit (Dojindo Chemical Laboratory, Kumamoto, Japan) was applied to measure the OD value of the cells at 24, 48, 72, and 96 hours. 100 *μ*l of free medium containing 10% CCK-8 solution was put into each well. After being cultured for 1-4 h in a humid incubator at 37°C and 5% CO_2_, the cells in 450 nm were detected.

### 2.9. Cell Migration and Invasion Assay

A 24-hole Transwell insertion chamber and an 8 *μ*m pore polycarbonate filter were used for migration analysis. 1 × 10^4^ cells were maintained in a fresh medium in the above chamber. A medium about 600 *μ*l containing 10% FBS was filled in the down chamber. In the invasion test, a matrix gel invasion chamber in a 24-well plate was used. 1 × 10^5^ cells were transplanted into the fresh medium in the upper chamber. 600 *μ*l of medium containing 10% FBS was put in the lower chamber. The cells got fixed with formaldehyde for 10 minutes and stained with DAPI for 15 minutes at 20-hour incubation. Averagely, five fields were stochastically chosen to calculate the amount of migrated or invaded cells under an inverted fluorescence microscope.

### 2.10. Statistical Analysis

SPSS17.0 (SPSS Corporation, Chicago, Illinois, USA) and GraphPad Prism 5 (GraphPad Software, Inc., La Jolla, CA, USA) were conducted to perform statistics analysis. *P* < 0.05 represented significant statistical difference. The differences existing in the two groups were analyzed by the Student *t*-test. The comparison of multiple groups was dissected by the Bonferroni post hoc test. One-way analysis of variance (ANOVA) was used for comparison of the difference of multiple groups. The survival rate of patients was assessed by Kaplan-Meier analysis.

## 3. Results

### 3.1. ROM1 Was a Promising Inhibitor Gene in Lung Cancer

**Figure 1:**
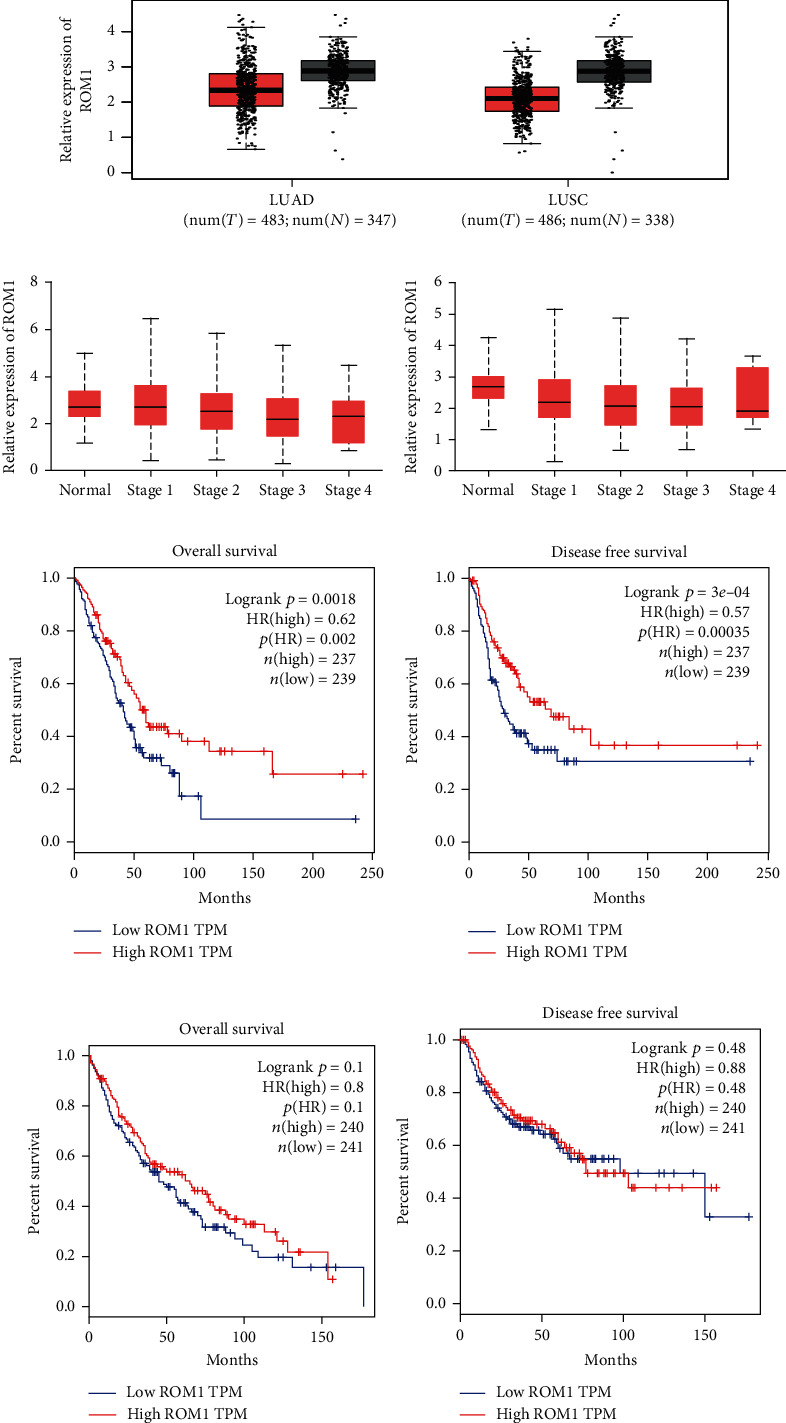
In lung cancer, ROM1 was downregulated, and the expression level is related to tumor stage and is associated with poor prognosis: (a, b) ROM1 expression in LUAD and LUSC in the TCGA cohort (^∗^*P* < 0.05 vs. normal) and its correlation with tumor stage; (d, e) Kaplan-Meier OS curve and DFS curve of LUAD patients (*n* = 476); (f, g) Kaplan-Meier OS curve and DFS curve of LUSC patients (*n* = 481).

For the study of ROM1 level in lung cancer, we firstly assessed RNA sequencing data in TCGA database. Our data revealed that compared to 347 normal tissues, ROM1 expression was greatly reduced in 483 LUAD tissues. Besides, compared with 338 normal tissues, ROM1 was also dramatically decreased in 486 LUSC tissues (Figure 1(a)). Among lung cancer patients, we further validated the association between ROM1 expression and prognostic status. Our results indicated that ROM1 level displayed correlation with the tumor stage in LUAD and LUSC (Figures 1(b) and 1(c)). Given the key value of ROM1 expression in LUAD, LUAD was then separated into highly expressed (*n* = 237) and lowly expressed groups (*n* = 239). Kaplan-Meier analysis revealed that patients with lowly expressed ROM1 demonstrated shorter overall survival (OS) time and disease-free survival (DFS) (Figures 1(d) and 1(e)). Besides, LUSC patients were then classified into highly expressed (*n* = 240) and lowly expressed groups (*n* = 241) based on the primary value of ROM1 expression in LUSC. Our data further indicated that there might be a correlation between ROM1 level represented and the OS time and DFS of LUSC patients (Figures 1(f) and 1(g)). Collectively, we assumed that ROM1 was a promising inhibitor gene for lung cancer.

### 3.2. Reduced ROM1 Indicated Poor Prognosis in LUAD and LUSC

**Figure 2:**
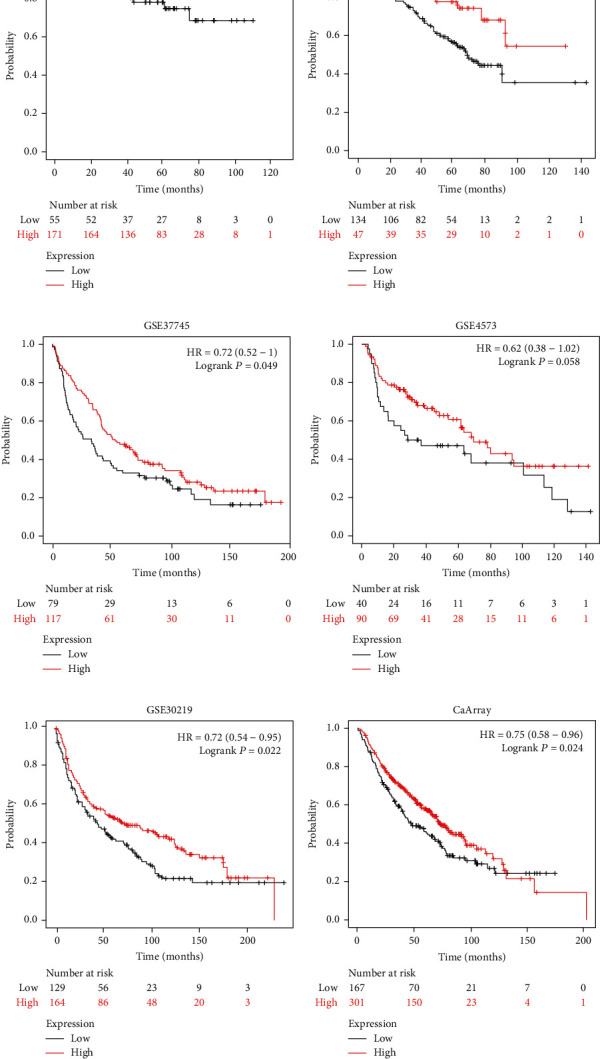
Kaplan-Meier analysis showed that the survival probability of patients in the ROM1 low expression group was shortened. Downloaded ROM1 expression profile data of lung cancer samples from the Gene Expression Omnibus (GEO) database included GSE31210 (a), GSE50081 (b), GSE37745 (c), GSE4573 d), GSE30219 (e), and cancer microarray informatics (CaArray) data management (f) and analysis of its association with survival rate.

We analyzed multiple databases to further validate the relationship between ROM1 expression level and survival rate in LUAD and LUSC. Our data suggested that in the GSE31210 (Figure 2(a)), GSE50081 (Figure 2(b)), and GSE37745 (Figure 2(c)) databases, Kaplan-Meier analysis revealed that the survival rate of LUAD patients in the highly expressed ROM1 group was dramatically higher relative to that in the lowly expressed ROM1 group. Additionally, we got a similar result towards ROM1 expression and the survival rate of LUSC in GSE4573 (Figure 2(d)), GSE30219 (Figure 2(e)), and CaArray (Figure 2(f)) databases.

### 3.3. GO Term and KEGG Pathway Analysis of Differentially Expressed Genes (DEGs)

**Figure 3:**
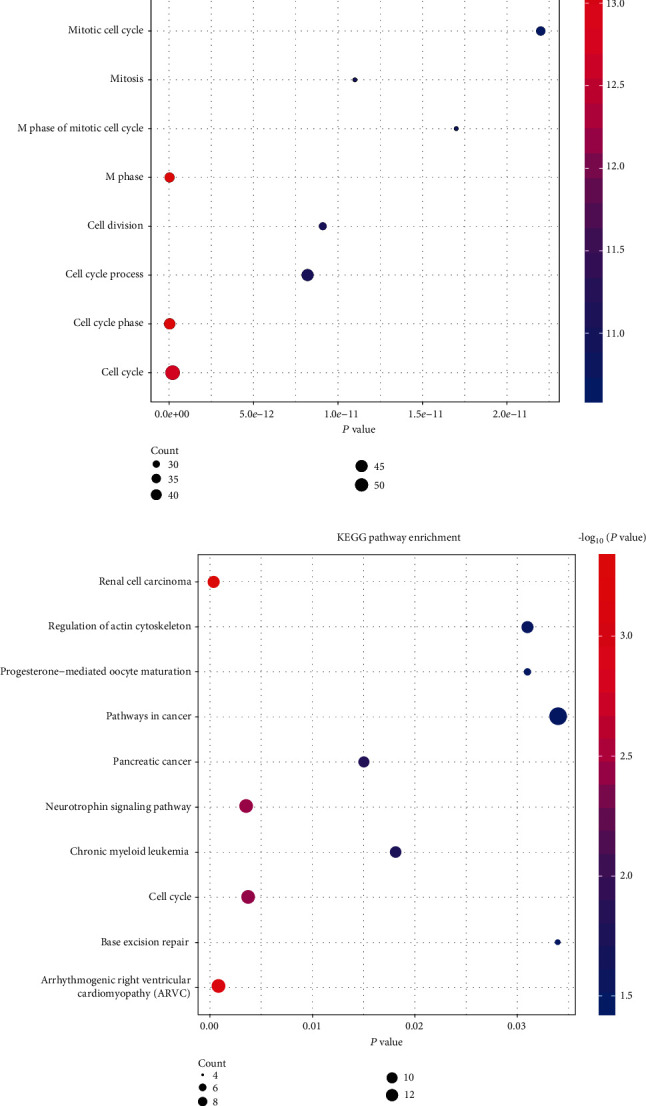
Analysis of GO term enrichment and KEGG pathways of DEGs in lung cancer: (a) GO term enrichment analysis of DEGs in lung cancer in the TCGA cohort; (b) KEGG pathways of DEGs in lung cancer in the TCGA cohort. Reactor pathways of significant DEGs are shown on the *y*-axis. The *x*-axis represents the DEGs of different groups. Different colors of the dots indicated different *P* values, and the different sizes of the dots illustrated the gene ratio of the corresponding GO term or KEGG pathway.

We primarily assessed the biological process (BP) through DAVID (Table 1) for further understanding the functions of these DEGs. For BP, the top ten pathways enriched were cell cycle and its phase and process, cell division, M phase, M phase of mitotic cell cycle, mitosis, mitotic cell cycle, nuclear division, and organelle fission (Figure 3(a)). The DEGs of KEGG pathway enrichment analysis data showed that the top ten KEGG pathways enriched were renal cell carcinoma, arrhythmogenic right ventricular cardiomyopathy (ARVC), base excision repair, cell cycle, chronic myeloid leukemia, neurotrophin signaling pathway, pancreatic cancer, pathways in cancer, progesterone-mediated oocyte maturation, and regulation of actin cytoskeleton (Table 2) (Figure 3(b)). All these significantly rich GO terms and approaches will be conducive to understanding the pivotal molecular mechanism involved in lung cancer development.

### 3.4. ROM1 Was Reduced in Lung Cancer and Cells

**Figure 4:**
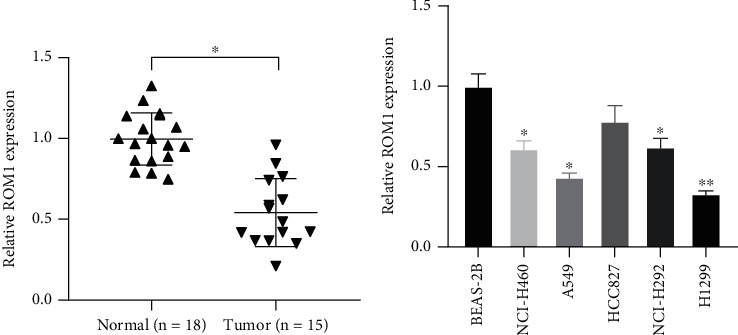
ROM1 was downregulated in lung cancer and cell lines: (a) ROM1 was reduced in the cancer tissues; (b) ROM1 was decreased in lung cancer cells. ^∗^*P* < 0.05; ^∗∗^*P* < 0.01.

In order to confirm TCGA database results, RT-qPCR was executed to detect ROM1 level in tissues of lung cancer and adjacent normal ones. Compared with 18 cases of normal tissues, ROM1 expression in 15 cases of lung cancer tumor tissues was significantly downregulated (Figure 4(a)). Additionally, ROM1 level in lung cancer cell lines was measured. In comparison with that in normal lung cells, ROM1 in lung cancer cells was largely lower (Figure 4(b)). The changes of ROM1 in cells and tissues were similar. Considering ROM1 expression level, we believed that ROM1 might function as lung cancer inhibitor.

### 3.5. Knockout of ROM1 Gene Promoted Lung Cancer Cell Proliferation

**Figure 5:**
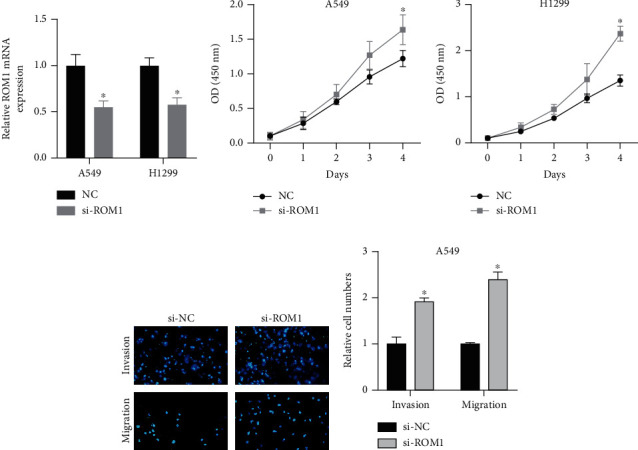
Knockdown of ROM1 gene promoted lung cancer cell proliferation, migration, and invasion. (a) ROM1 expression was reduced in siRNA-transfected A549 and H1299 cells. (b, c) CCK-8 assay detection of cell proliferation of above-treated cells. (d, e) Knockdown of ROM1 suppressed A549 cells in migrating, invading, and the result number. ^∗^*P* < 0.05; ^∗∗^*P* < 0.01.

Reduced ROM1 level was both shown in lung cancer tissues and cells (Figures 4(a) and 4(b)). ROM1 probably participated in the modulation of cell proliferation. ROM1 was knocked down by siRNAs and further evaluated its role in lung cancer. qRT-PCR data revealed that ROM1 expression level was greatly reduced in A549 and H1299 cells (Figure 5(a)). CCK-8 assay deeply demonstrated that ablated ROM1 induced cell proliferation, indicating that ROM1 modulated lung cancer cell proliferation (Figures 5(b) and 5(c)).

### 3.6. Knockout of the ROM1 Gene Promoted Cell Migration and Invasion of Lung Cancer

Transwell experiments were performed to validate the impacts of ROM1 on lung cancer cell invasion and migration. Transwell analysis results indicated that a significantly increased number of migrated and invaded lung cancer cells were demonstrated in ROM1 knockdown group (Figures 5(d) and 5(e)). Generally, ROM1 exhibited importance in regulating cell migration and invasion.

## 4. Discussion

Biological characteristics reveal that lung cancer comprises non-small-cell lung cancer and small cell lung cancer (SCLC). NSCLC takes up about 85-90% over all lung cancer [16]. LUAD and LUSC account for a higher proportion [17]. As far as NSCLC treatment is concerned, genetic testing is conducted and optimal targeted drugs can be chosen [18, 19]. It is more and more obvious that the functional importance of mRNA in tumor biology has attracted focus. Usually, it is verified that mRNAs can be a downstream target in cancer progression [20, 21]. Several researches suggested that some mRNAs participated in tumorigenesis process by modulating gene expression. For instance, Leibovitch and Topisirovic explored the dysregulation of mRNA translation and energy metabolism in cancer [22]. Therefore, to find suitable biomarkers and therapeutic targets for NSCLC patients' treatment and prognosis is urgently needed.

Here, lung cancer-related data sets were analyzed. GO analysis of DEGs showed that these overlapping DEGs were mostly related to cell cycle and cell cycle phase. Additionally, KEGG pathway enrichment analysis demonstrated that these overlapping DEGs were greatly rich in arrhythmogenic right ventricular cardiomyopathy (ARVC), cell cycle, pancreatic cancer, and pathways in cancer. These rich gene functions and KEGG pathway provide ideas for understanding the molecular mechanism of lung cancer.

The retinal outer segment membrane protein is abnormally expressed in many cancers, but the mechanism of ROM1 gene in lung cancer is unknown. As far as we know, this is the first report on the progress of ROM1's involvement in NSCLC. Our study firstly assessed ROM1 expression in lung cancer tissues utilizing RNA sequencing data from TCGA database. ROM1 was reduced in lung cancer tissues, and its expression level represented a correlation with tumor stages in LUAD and LUSC. Downregulation of ROM1 displayed an association with the poorly prognostic status of lung cancer patients. The results indicated that ROM1 was a probably diagnostic biomarker and was represented as an inhibited gene for lung cancer.

We also explored how ROM1 influenced lung cancer cells in proliferation, migration and invasion. Notably, the imbalance of cell proliferation was a key point of affecting cancer tumorigenesis and development. We found that knocking out ROM1 significantly promoted lung cancer cell proliferation. Cell invasion and metastasis are important for the prognostic profile of lung cancer patients. The Transwell test showed that knocking out ROM1 significantly promoted lung cancer cell migration and invasion. Thus, ROM1 was perhaps involved in lung cancer development by modulating cell proliferation, invasion, and metastasis. In future studies, we will collect more samples to study the correlation between ROM1 expression and clinical parameters (including clinical stage, age, and survival time).

In conclusion, our data reveal that lung cancer possesses reduced ROM1 and is reported to be related to the tumor stage and prognosis, indicating that ROM1 plays importance in lung cancer. Our study collectively indicates that ROM1 is beneficial for lung cancer diagnosis and treatment.

## Figures and Tables

**Table 1:** GO analysis of biological process of DEGs associated with lung cancer.

Category	Term	RT	Count	%	*P* value	Benjamini
GOTERM_BP_ALL	M phase	RT	35	8.4	3.00*E*-14	5.30*E*-11
GOTERM_BP_ALL	Cell cycle phase	RT	39	9.3	3.80*E*-14	3.40*E*-11
GOTERM_BP_ALL	Organelle fission	RT	29	6.9	9.70*E*-14	5.70*E*-11
GOTERM_BP_ALL	Cell cycle	RT	53	12.6	2.10*E*-13	9.10*E*-11
GOTERM_BP_ALL	Cell cycle process	RT	42	10	8.20*E*-12	2.90*E*-09
GOTERM_BP_ALL	Cell division	RT	30	7.2	9.10*E*-12	2.70*E*-09
GOTERM_BP_ALL	Nuclear division	RT	26	6.2	1.10*E*-11	2.80*E*-09
GOTERM_BP_ALL	Mitosis	RT	26	6.2	1.10*E*-11	2.80*E*-09
GOTERM_BP_ALL	M phase of mitotic cell cycle	RT	26	6.2	1.70*E*-11	3.70*E*-09
GOTERM_BP_ALL	Mitotic cell cycle	RT	33	7.9	2.20*E*-11	4.40*E*-09

**Table 2:** KEGG pathway enrichment of these DEGs.

Category	Term	RT	Count	%	*P* value	Benjamini
KEGG_PATHWAY	Renal cell carcinoma	RT	8	1.9	5.10*E*-04	6.00*E*-02
KEGG_PATHWAY	Arrhythmogenic right ventricular cardiomyopathy (ARVC)	RT	8	1.9	8.40*E*-04	5.00*E*-02
KEGG_PATHWAY	Neurotrophin signaling pathway	RT	9	2.1	3.60*E*-03	1.40*E*-01
KEGG_PATHWAY	Cell cycle	RT	9	2.1	3.80*E*-03	1.10*E*-01
KEGG_PATHWAY	Pancreatic cancer	RT	6	1.4	1.50*E*-02	3.10*E*-01
KEGG_PATHWAY	Chronic myeloid leukemia	RT	6	1.4	1.80*E*-02	3.10*E*-01
KEGG_PATHWAY	Progesterone-mediated oocyte maturation	RT	6	1.4	3.10*E*-02	4.20*E*-01
KEGG_PATHWAY	Regulation of actin cytoskeleton	RT	10	2.4	3.10*E*-02	3.80*E*-01
KEGG_PATHWAY	Base excision repair	RT	4	1	3.40*E*-02	3.70*E*-01
KEGG_PATHWAY	Pathways in cancer	RT	13	3.1	3.40*E*-02	3.50*E*-01

## Data Availability

The data sets used and/or analyzed during the current study are available from the corresponding author on reasonable request.

## Abbreviations

NSCLC: Non-small-cell lung cancer
MD/PD: Macular/pattern dystrophy
RP: Retinitis pigmentosa
ccRCC: Clear cell renal cell carcinoma
ROM1: Outer retinal membrane protein 1
ATCC: American Type Culture Collection
siRNA: Small interfering RNA
qRT-PCR: Quantitative reverse transcription PCR
CCK-8: Cell counting 8 kit
TCGA: Cancer Genome Atlas
LUAD: Lung adenocarcinoma
LUSC: Lung squamous cell carcinoma
GEO: Gene Expression Omnibus
CaArray: Cancer microarray
GSEA: Gene Set Enrichment Analysis
GO: Gene ontology
KEGG: Kyoto Encyclopedia of Genes and Genomes
BP: Biological process
ARVC: Arrhythmogenic right ventricular cardiomyopathy
SCLC: Small cell lung cancer
OS: Overall survival
DFS: Disease-free survival.
